# Epigenetic Regulation and Functional Characterization of MicroRNA-142 in Mesenchymal Cells

**DOI:** 10.1371/journal.pone.0079231

**Published:** 2013-11-13

**Authors:** Magne Skårn, Tale Barøy, Eva Wessel Stratford, Ola Myklebost

**Affiliations:** 1 Department of Tumor Biology, Institute for Cancer Research, The Norwegian Radium Hospital, Oslo University Hospital, Oslo, Norway; 2 Cancer Stem Cell Innovation Centre, Institute for Cancer Research, The Norwegian Radium Hospital, Oslo University Hospital, Oslo, Norway; 3 Norwegian Stem Cell Centre, Oslo University Hospital, Oslo, Norway; Université Paris-Diderot, France

## Abstract

The transcripts encoded by the microRNA *mir-142* gene are highly active in hematopoietic cells, but expressed at low levels in many other cell types. Treatment with the demethylating agent 5-Aza-2′-deoxycytidine increased both the 1,636 nucleotide primary transcript and mature miR-142-5p/3p in mesenchymal cells, indicating that *mir-142* is epigenetically repressed by DNA methylation. The transcription start site was determined to be located 1,205 base pairs upstream of the precursor sequence within a highly conserved CpG island. In addition, a second CpG island overlapped with the precursor. A TATA-box, several promoter-proximal elements and enrichment of conserved transcription factor binding sites within the first 100 base pairs upstream of the transcription start site, suggests that this region represents the core/proximal *mir-142* promoter. Moreover, both CpG islands were heavily methylated in mesenchymal cells, having low levels of miR-142-5p/3p, and unmethylated in hematopoietic cells where both miRNAs were abundantly expressed. We show that treatment with 5-Aza-2′-deoxycytidine significantly reduced the DNA methylation of the upstream CpG island, which led to increased expression, and that *in vitro* DNA methylation of the upstream region of the *mir-142* precursor repressed its transcriptional activity. When overexpressed, miR-142-5p/3p reduced proliferation of cells with epigenetic silencing of endogenous *mir-142*. This finding is interesting as miR-142-5p/3p have been reported to be deregulated in tumors of mesenchymal origin. We provide the first experimental evidence that transcription of *mir-142* is directly repressed by DNA methylation. In addition, we discovered that the antisense strand of *mir-142* might act as a precursor for functional mature antisense miRNAs. Thus, our study expands the current knowledge about the regulation of *mir-142* and function of miR-142-5p/3p, and adds novel insight into the rapidly increasing field of microRNA regulation.

## Introduction

microRNAs (miRNAs) are short endogenous non-coding RNAs (ncRNAs) that inhibit the expression of target genes at the post-transcriptional level [1]. In mammals, miRNAs are estimated to regulate 30–60% of protein-coding genes [2], [3]. Most miRNA genes are transcribed as mono- or polycistronic stem-loop structures by RNA polymerase II [4]. Among 939 human miRNAs analyzed by Sato et al., 31% were intergenic (i.e. located between annotated protein-coding genes), whereas the remaining were intragenic; localized within either protein-coding or ncRNA transcripts in either sense (44%), antisense (13%) or both orientations (12%) [5]. The intergenic miRNAs are independent transcription units, with their own regulatory elements [4], whereas intragenic miRNAs are co-transcribed with their host genes [6], or have their own promoters [7], [8]. In addition, many miRNA loci contain putative transcription units on both DNA strands. Transcription and processing of the strand antisense to an annotated miRNA have been reported in several studies from *Drosophila* [9]–[11] and mammals [12], [13].

Mature miRNAs are generated from longer primary miRNA transcripts (pri-miRNAs) [14], through an intermediate precursor (pre-miRNA) [15], [16], which has a length of approximately 70 nucleotides (nt) and adopts a characteristic stem-loop structure with a 2 nt 3′- overhang [17]. The mature miRNA, which in most cases represents one strand of the precursor, is incorporated into the miRNA-induced silencing complex (miRISC). Guided by the miRNA, miRISC targets specific messenger RNAs, leading to transcriptional destabilization and/or post-transcriptional inhibition (reviewed in [18]). miRNAs are typically expressed in cell-, tissue- and development-specific manners [19]–[25]. Evidence is emerging that similar to protein-coding genes, epigenetic mechanisms such as DNA methylation and post-translational modifications of histones are involved in the regulation of miRNA expression [25]. DNA methylation at the dinucleotide CpG is one of the most common epigenetic modification in eukaryotic genomes, and plays a crucial role in various biological processes [26]. CpG islands (CGIs) are GC-rich regions with high relative densities of CpGs [27], which colocalize with the promoter and transcription start site (TSS) of approximately 50–70% of human proteins-coding genes [28]. The number could be similar for miRNAs, as 155 out of 332 human miRNA genes have been found to be associated with CGIs [29]. DNA methylation of CGIs often correlates with repression of transcription as a result of decreased affinity for transcription factors (TFs) [30] and/or by facilitating the assembly of chromatin-modifying repressor complexes at the methylated regions [31]. Besides DNA methylation, various histone modifications act as positive or negative regulators of gene expression (reviewed in [32]). Examples of permissive marks are trimethylation of lysine 4 on histone H3 (H3K4me3), dimethylation of lysine 79 on histone H3 (H3K79me2) and acetylation of histone H3 at various lysine residues, e.g. H3K27ac. Examples of repressive marks are H3K9me3 and H3K27me3.

*mir-142* was first identified as hematopoietic-specific by tissue-specific cloning of short RNAs [22]. Its encoded transcripts are expressed in various hematopoietic cell types and virtually absent elsewhere [19], [23], [33]. *mir-142* plays a crucial role in immune cell development and –function [19], [34]–[39]. Moreover, *mir-142* has been reported to be regulated at the genetic level by mutations in the precursor sequence (*pre-mir-142*) [36], at the transcriptional level by LIM domain only 2 (LMO2) [40] and at the post-transcriptional level by extensive RNA editing [41]. Furthermore, reduced expression of miR-142-5p/3p in systemic lupus erytematosus CD4 molecule (CD4) positive T cells has been associated with changes to DNA methylation levels and histone modifications upstream of *pre-mir-142* [34]. Hematopoietic stem cells mainly reside in the bone marrow, where their function is tightly regulated by the microenvironment (reviewed in [42]). Non-hematopoietic cells, such as multipotent human mesenchymal stromal cells (also known as human mesenchymal stem cells, hMSCs) and osteoblasts constitute essential components of the niche [43]. These cell types express very low levels of miR-142-5p/3p transcripts. However, the mechanism responsible for this repression has not previously been investigated.

In the present study, the objective was to determine why miR-142-5p/3p are expressed at low levels in mesenchymal cells. To this end we performed a detailed *in silico* and *in vitro* characterization of the *mir-142* locus and its encoded transcripts. We suggest that transcription of *mir-142* in mesenchymal cells is repressed by DNA methylation of an upstream conserved promoter-associated CGI, and that the cell type-specific expression of *mir-142* could be predominantly determined by methylation. Furthermore, ectopic expression of miR-142-5p/3p revealed that they are involved in the regulation of proliferation, and possibly also migration and colony forming ability of mesenchymal cells. Finally, our results showed that transcription and processing of the antisense strand of the *mir-142* locus potentially can give rise to functional mature miRNAs, which have previously not been described.

## Materials and Methods

### Cell culture and RNA

Eight osteosarcoma cells lines were analyzed. The OHS cell line was established at the Norwegian Radium Hospital, whereas four other cell lines (IOR/OS14, IOR/OS10, IOR/SARG and IOR/MOS) were established at the the Istituto Ortopedico Rizzoli. The cell lines HOS, U-2 OS, MG-63 as well as the K562 leukemia cells were obtained from American Type Culture Collection (http://www.lgcstandards-atcc.org). The osteosarcoma cells and the K562 cells were cultured in RPMI 1640 and DMEM (both from Life Technologies, Carlsbad, USA), respectively, and supplemented with 10% fetal bovine serum (PAA Laboratories, Pasching, Austria), GlutaMAX (2 mM), penicillin (100 U/ml) and streptomycin (100 µg/ml) (all from Life Technologies) at 37°C and 5% CO_2_. The primary hMSCs were isolated and cultured as previously described [44], and the primary osteoblasts (Sciencell Research Laboratories, Carlsbad, USA) were cultured as described by the manufacturer. The immortalized mesenchymal cell line iMSC#3 has been previously described [45]. The peripheral blood progenitor cells (PBPCs) from a healthy donor were mobilized using recombinant human granulocyte colony-stimulating factor and collected by leukapheresis using standard methods. The brain RNA was purchased from Life Technologies (Cat. No. AM6050).

### Ethics statement

The primary hMSCs and PBPCs were obtained from healthy donors after written consent. The informed consent and sample collection were approved by the Regional Ethical Committee for Southern Norway (S-90128).

### 5-Aza-2′-deoxycytidine and trichostatin A treatment

The cell lines MG-63, OHS, IOR/OS14, U-2 OS, IOR/OS10, IOR/SARG, IOR/MOS, HOS, iMSC#3 and K562 were subjected to treatment with 1 µM of the demethylating drug 5-Aza-2′-deoxycytidine (5-Aza) (Cat. No. A3656, Sigma-Aldrich, St. Louis, USA) for 3 days (5 days for iMSC#3), as previously described [46]. MG-63 cells were also treated with a combination of 1 µM of 5-Aza and 0.5 µM of the histone deacetylase inhibitor trichostatin A (TSA) (Cat. No. T8552, Sigma-Aldrich), added the last 12 hours, or 0.5 µM of TSA alone for 12 hours.

### *In silico* analyses

Three different algorithms were used to search for CGIs in the ±5 kilobases flanking sequences of *pre-mir-142*. The CpG Island Searcher is based on the criteria established by Takai and Jones, which defines a CGI as regions of DNA greater than 500 base pairs (bp) with a G+C content ≥55% and an observed CpG/expected CpG ratio ≥65% [47]. The CpG Island Explorer, a modification of the former algorithm defines a CGI as a region of length ≥500 bp, G+C content ≥50% and observed CpG/expected CpG ratio ≥60%. The CpGcluster predicts statistically significant clusters of CpG dinucleotides, independently of the above criteria [48].

The University of California and Santa Cruz Genome Browser (UCSC Genome Browser) [49]was used to perform multiple alignments and to identify evolutionary conserved regions using the Vertebrate Multiz Alignment & Conservation (46 Species) track. The TFBS Conserved track was used to identify conserved TF-binding sites and the ENCODE Transcription Levels Assayed by RNA-seq on 6 Cell Lines track was used to find evidence to support the 5′- and 3′- boundaries of the primary *mir-142* transcript (*pri-mir-142*) [50]. The ENCODE TFBS tracks from Stanford/Yale/USC/Harward and HAIB were used to identify binding of TFs as determined by ChIP-seq [51]. The ENCODE/Broad histone modification by ChIP-seq track was used to map the *mir-142* locus in K562 cells and osteoblasts [52]. The ENCODE/HAIB CpG Methylation by Methyl 450K Bead Array track was used to analyze methylation level at *mir-142* locus in 60 cell lines. These data were produced at the HudsonAlpha Institute for Biotechnology.

Poly(A) Signal Miner was used to search for hexamers associated with termination of transcription [53], and the basic local alignment search tool was used to map various sequences to the human genome [54]. Positions are indicated relative to the 5′- end of *pre-mir-142*. All *in silico* analyses were performed using the human reference genome assembly GRCh37/hg19.

### Cloning of the *mir-142* gene

The AccuPrime Taq DNA Polymerase High Fidelity (Life Technologies) was used to amplify a 334 bp fragment consisting of *pre-mir-142* with flanking sequences, from human genomic DNA (6152A 19759702, Promega, Madison, USA). The amplicon was T/A-cloned into the CMV-driven expression construct pMaNeo/TO in the sense and antisense orientation (designated pCMV/*mir-142* and pCMV/*mir-142*_AS, respectively), essentially as described in [44] (Table S1 for sequences). All constructs were sequenced to verify the correct insert.

### Quantitative real-time PCR

Quantitative real-time PCR (qPCR) was performed using the ABI PRISM 7500 DNA Sequence Detection System (Life Technologies). The relative expression levels were determined using the comparative threshold cycle (2^−ΔΔCT^) [55]. Total RNA was isolated using TRIzol Solution (Life Technologies) and treated with amplification-grade DNase I (Life Technologies). The High Capacity RNA-to-cDNA Master Mix (Life Technologies) was used to synthesize cDNA. The ProbeFinder and Universal ProbeLibrary Assays (Roche Applied Science, Indianapolis, USA) were used to investigate the transcriptional activity at the *mir-142* locus. Assays designed to be specific for four different upstream regions (Table S1) were used to identify the approximate TSS of *mir-142*. Universal ProbeLibrary Assays and probes were also used to quantify expression of the benzodiazapine receptor associated protein 1 antisense RNA 1 (*BZRAP1-AS1*). The assays were designed to span exon/intron boundaries (Table S1). Glyceraldehyde 3-phosphate dehydrogenase (*GAPDH*) was used to normalize the data (Life Technologies) (Table S2). The TaqMan MicroRNA RT Kit and various TaqMan MiRNA Assays (Life Technologies) (Table S2) were used to quantitatively detect mature miRNAs. The *RNU44* (small nucleolar RNA, C/D box 44) was used for normalization. The error bars in all qPCR experiments show the standard deviation of technical replicates, calculated as previously described [56].

### 5′- and 3′- rapid amplification of cDNA ends

The SMARTer RACE cDNA Amplification Kit (Clontech, Mountain View, USA) was used to perform both 5′- and 3′-rapid amplification of cDNA ends (RACE). Polyadenylated RNA was isolated using the Oligotex Direct mRNA Mini Kit (Qiagen, Hilden, Germany) and the gene-specific primers (GSPs) were designed using Primer3Plus [57] (Table S3). The Advantage 2 Polymerase Mix (Clontech) was used for the primary 2-step touchdown PCR, whereas the DNR Taq Polymerase (Kindly provided by Dave Warren at the Norwegian Radium Hospital) was used for the nested PCR. The amplification products were cloned using the TOPO TA Cloning Kit for sequencing (Life Technologies) and several clones were sequenced.

### Bisulfite treatment of DNA, methylation-specific polymerase chain reaction and bisulfite sequencing

Genomic DNA isolated using the Wizard Genomic DNA Purification Kit (Promega) was bisulfite treated using the EpiTect Bisulfite Kit (Qiagen) and purified using the Qiacube (Qiagen). Primers were designed using MethPrimer [58] or Methyl Primer Express (Life Technologies) (Table S4). The 2 methylation-specific PCR (MSP) sets were designed to determine the methylation status of 5 CpGs located approximately 1,300 bp upstream (MSP #1) and of 5 CpGs in the immediate flanking regions of the *pre-mir-142* (MSP #2). Approximately 22.8 ng of bisulfite converted DNA was used as input for the EpiTect MSP Kit, and the EpiTect PCR Control DNA Set (Both from Qiagen) was used for standardized optimization and control experiments (Figure S1). The PCR products were separated by electrophoresis using a 2% agarose gel.

For the bisulfite sequencing, approximately 20 ng of bisulfite modified DNA was amplified, using the AccuPrime Taq DNA Polymerase High Fidelity (Life Technologies). The conditions and primers used for the semi-nested PCR were the same as those for the primary PCR, except that buffer I was used instead of buffer II. A 368 bp fragment between −1,483 to −1,115 containing 18 CpGs (region #1), and a 366 bp fragment between −95 to +271, containing 14 CpGs (region #2) (positions relative to 5′- end of *pre-mir-142*) were amplified. The amplicons were purified using the Wizard SV Gel and PCR Clean-Up System, subsequently adenosine-tailed and cloned into pGEM-T Easy (Promega), before being subjected to sequencing. The CpGviewer was used to analyze the resulting sequences [59].

### Cloning of the upstream region of precursor *mir-142*, construction of expression constructs, *in vitro* DNA methylation and luciferase experiments

The 2,031 bp upstream region of *pre-mir-142* was amplified using PCR and The AccuPrime Taq DNA Polymerase High Fidelity (Life Technologies) (Table S5) and T/A-cloned into the CpG-free pCpGL-basic luciferase reporter construct, which was a gift from Michael Rehli [60]. To allow T/A cloning, the vector was digested with *Hin*dIII and *Pst*I (both from New England Biolabs), blunted using T4 DNA polymerase (New England Biolabs) and subsequently thymidine-tailed. The pCpGL-basic with the cloned upstream region was designated pCpGL/2031. 18 µg of pCpGL/2031 were *in vitro* methylated using the CpG methyltransferase M.SssI (4 units/µg), in the presence of 640 µM S-Adenosylmethionine (both from New England Biolabs) for 3 hours at 37°C and designated pCpGL/2031_M.SssI. The unmethylated control reporter vector (pCpGL/2031_mock) was treated as above but without methyltransferase. The constructs were purified using the Wizard SV Gel and PCR Clean-Up System (Promega), and methylation of the insert was confirmed using *Hpa*II (New England Biolabs) (data not shown). The resulting constructs were co-transfected with the *Renilla* luciferase vector pRL-TK (Promega) into U-2 OS using the the ExGen 500 *in vitro* Transfection Reagent (Cat. No. #R0511, Thermo Fisher Scientific, Waltham, USA). Briefly, 2,000–4,000 cells per well were plated in standard 96-well plates approximately 16 hours before transfection with 5 equivalents of ExGen 500 per well (i.e. 0.8 µl transfection reagent, 260 ng *Firefly* luciferase reporter and 40 ng of the *Renilla* luciferase reporter. Luciferase activities were measured 48 hours after transfection using the Dual-Luciferase Reporter Assay System and the Modulus Microplate Multimode Reader (Both from Promega). The experiments were performed in ≥ quadruplicate wells in ≥5 biological replicates (n≥5). Luciferase activity was defined as the ratio between *Firefly* luciferase- and *Renilla* luciferase activity.

### Ectopic expression of miRNAs in osteosarcoma cells

The cells were seeded at a density of 625–1,250 cells per well in standard 96-well plates the day before transfection. Synthetic miRNA mimics representing miR-142-5p/3p and a negative control (Life Technologies) (Table S2) were transiently transfected into MG-63 cells either individually or together, using the INTERFERin siRNA transfection reagent (Polyplus-transfection SA, Illkirch, France). Briefly, the synthetic miRNA was diluted with Opti-MEM (Life Technologies) and mixed with 0.8 µl of INTERFERin to give a final concentration of 15 nM in a total volume of 125 µl per well. The pCMV/*mir-142*, pCMV/*mir-142*_AS constructs and empty vector control (pCMV) were stably transfected into MG-63 cells using Lipofectamine 2000 (Life Technologies). Cells were selected for 2 weeks using 400 µg/ml of Geneticin/G418 sulfate (Cat. No. 10131-035, Life Technologies). The same constructs were also transiently transfected into OHS cells using the ExGen 500 *in vitro* Transfection Reagent (Thermo Fisher Scientific).

### Live cell imaging

The cellular proliferation rates after transfection was determined by live cell imaging using the IncuCyte Kinetic Imaging System (Essen BioScience, Hertfordshire, United Kingdom). Cell confluence of ≥5 replicate wells was determined every second hour for the duration of the experiment, and ≥2 biological experiments were performed (n≥2).

### Migration and colony forming assays

The migration and colony forming assays for the stably transfected MG-63 cells were performed as previously described [61].

### Statistical analysis

The non-parametric Wilcoxon signed rank test and the SPSS version 20 software (SPSS, Chicago, USA) was used to test for statistical differences. A *P* value ≤0.05 was considered to be significant and *n* indicates the number of experiments. To test for statistical differences between cells treated with 5-Aza and untreated cells, the ratio of methylated CpGs to the total number of CpGs was used. Region #1 and #2 were tested independently and together, as well as the CpG-site associated with the enhancer box (E-box) in region #1 and the first 3 CpGs in region #2. This was done for all the clones, cells treated with 5-Aza and untreated cells as one group, and cells treated with 5-Aza as the other group. Clones where the status of all CpGs were not determined, was excluded from the analysis when region #1 and #2 were tested, but included when the specific CpGs were tested. Furthermore, the Wilcoxon signed rank test was used to test for statistical differences in the promoter activity and *in vitro* DNA methylation experiments of the upstream *pre-mir-142* region, as well as for the proliferation, migration and colony forming assays.

## Results

### *mir-142* transcripts are expressed at low levels in mesenchymal cells

**Figure 1:**
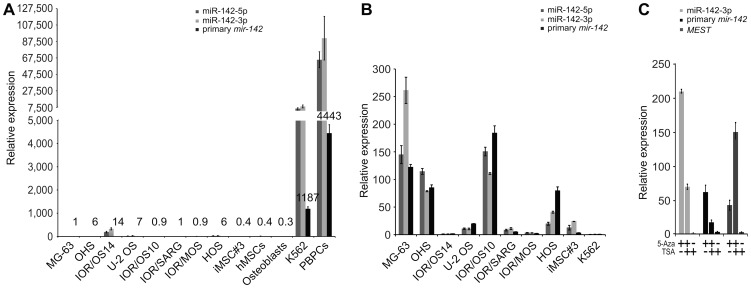
Endogenous transcript levels of mature miR-142-5p/3p and primary *mir-142* in cells of mesenchymal and hematopoietic origin, and after treatment with epigenetic modifiers. (**A**) Expression levels as determined by qPCR in tumorigenic cells (MG-63, OHS, IOR/OS14, U-2 OS, IOR/OS10, IOR/SARG, IOR/MOS and HOS), non-tumorigenic mesenchymal cells (iMSC#3, hMSCs and primary osteoblasts), and hematopoietic cells (K562 and PBPCs). Expression is depicted relative to the MG-63 cells (set to 1) and the numbers above the histograms represent the expression level of primary *mir-142*. *RNU44* or Glyceraldehyde 3-phosphate dehydrogenase (*GAPDH*) were used for normalization of mature and primary transcripts, respectively. iMSC#3, immortalized bone marrow-derived stromal cells; hMSCs, primary bone marrow-derived stromal cells; K562, K562 leukemia cells; PBPCs, peripheral blood progenitor cells. (**B**) Quantification of expression using qPCR after treatment with 5-Aza-2′-deoxycytidine (5-Aza). Expression is shown relative to untreated cells (set to 1). *RNU44* or *GAPDH* were used for normalization of mature and primary transcripts, respectively. (**C**) Expression levels of miR-142-3p, primary *mir-142* and mesoderm specific transcript (*MEST*) in MG-63 cells as determined by qPCR, after treatment with 5-Aza alone, a combination of 5-Aza and trichostatin A (TSA), or TSA alone. *RNU44* or *GAPDH* were used for normalization of mature and primary transcripts (including *MEST*), respectively. The error bars in all qPCR experiments show the standard deviation of technical replicates.

We have previously shown that miR-142-3p is repressed in tumorigenic cell lines of mesenchymal origin [62]. To confirm and expand these data, the transcript levels of both *pri-mir-142* and mature miR-142-5p/3p were quantified in 8 osteosarcoma cell lines using qPCR. In addition, we determined their expression in various non-tumorigenic mesenchymal cell types, such as primary and immortalized hMSCs (iMSC#3) and primary osteoblasts. Finally, we quantified their levels in K562 leukemia- and peripheral blood progenitor cells (PBPCs), which are cells of hematopoietic origin. In all cells of mesenchymal origin we found low levels of *pri-mir-142* and miR-142-5p/3p, whereas they were highly expressed in the hematopoietic cells (Figure 1A).

### Induction of *mir-142* in mesenchymal cells by treatment with 5-Aza

We suspected that *mir-142* was repressed by an epigenetic mechanism in mesenchymal cells. To address this, we treated iMSC#3 and various tumorigenic cell lines of mesenchymal origin, and also the K562 leukemia cells, with the demethylating agent 5-Aza. After 72 hours, the levels of miR-142-5p/3p were determined using qPCR. Increased expressions of both mature miRNAs (≥2-fold) were observed in all of the mesenchymal cell lines, except IOR/OS14 (Figure 1B). In the K562 cells, already having high levels, 5-Aza treatment had no effect. To determine if the increased levels of miR-142-5p/3p were mediated by upregulation of transcription, we quantified the level of *pri-mir-142*, which was induced coordinately (Figure 1B). In addition, the MG-63 cells were treated with 5-Aza combined with the histone deacetylase inhibitor TSA, or TSA alone (Figure 1C). The effect of the combination treatment on induction of *pri-mir-142* and miR-142-3p was reduced compared with that of 5-Aza alone, whereas treatment with only TSA had no impact on their expression. On the other hand, the expression of mesoderm specific transcript (*MEST*, i.e the host gene of *mir-335*), whose expression recently has been shown to be modified by TSA [63], showed the expected additive increase in cells treated with both epigenetic modifiers (approximately 150-fold), compared with an approximately 45-fold increase obtained using only 5-Aza. TSA alone did not affect the expression of *MEST*.

### *In silico* identification of putative CGIs associated with *mir-142*

**Figure 2:**
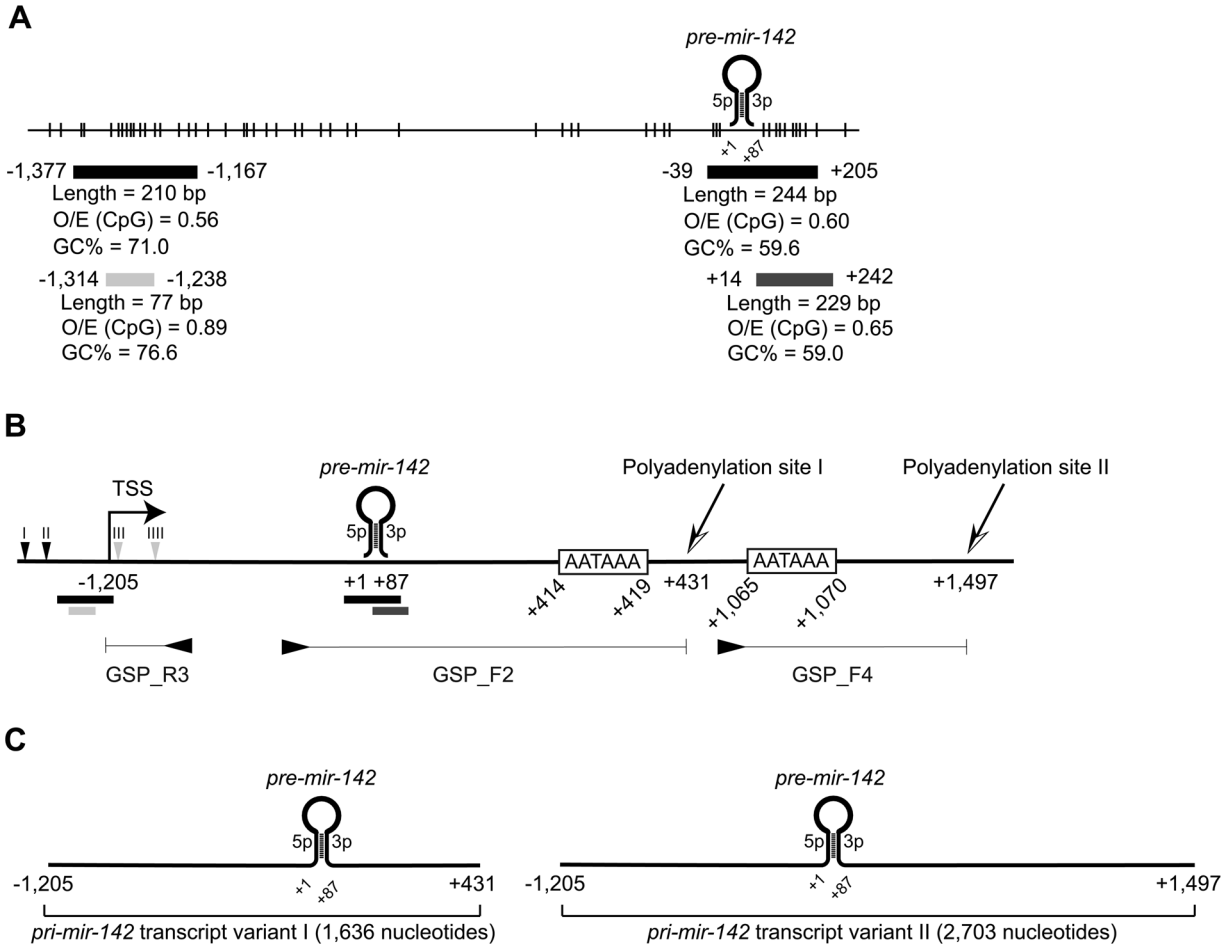
*In silico* identification of putative CGIs and characterization of the primary *mir-142* transcript. (**A**) Schematic representation of putative CGIs associated with *mir-142*. Each vertical line represents an individual CpG. Black, dark grey and light grey horizontal bars; CGIs predicted with CpG Island Explorer, CpG Island Searcher and CpGcluster, respectively. The numbers indicate the position relative to the *mir-142* precursor (*pre-mir-142*) (+1 to +87). O/E, observed/expected. (**B**) Schematic representation of the genomic *mir-142* region. The approximate 5′- end of *mir-142* was mapped using qPCR with assays specific for four different regions (depicted by vertical arrowheads). Grey and black arrowheads indicate increased expression or no change, respectively. The transcription start (TSS) and polyadenylation sites were identified using rapid amplification of cDNA ends. The approximate positions of the gene-specific primers (GSPs) and associated amplicons are indicated by horizontal arrowheads and attached lines, respectively. The putative CGIs are represented by horizontal bars. (**C**) Schematic representation of the 2 transcript variants of primary *mir-142* (*pri-mir-142*).

The concomitant increase of both primary and mature transcripts after inhibition of DNA methylation suggested that transcription was initiated from a promoter-associated CGI. To address this, we searched for putative CGIs within 5 kb up- and downstream of the *pre-mir-142* sequence using 3 different algorithms (Figure 2A). No CGIs were found using CpG Island Searcher and CpG Island Explorer [47] at the default settings, whereas the CpGcluster predicted a 77 bp long CGI located −1,314 to −1,238, relative to the 5′- end of *pre-mir-142*. When using less stringent criteria, the CpG Island Explorer identified a putative 210 bp CGI which overlapped with the CGI identified by CpGcluster. A 244 bp CGI between −39 to +205 was also predicted, which was also identified by CpG Island Searcher when the length requirement was reduced to ≥200 bp. Accordingly, this suggested that also *pre-mir-142* could be embedded in a CGI.

### Molecular characterization of the *pri-mir-142* transcript

Next, we wanted to determine if the transcription start of *mir-142* was associated with the upstream CGI. First we mapped the 5′- end to a region 1 to 1.6 kb upstream of *pre-mir-142*, by comparing the transcript levels in MG-63 cells treated or not treated with 5-Aza using four different qPCR assays (Figure 2B and Table S1). We further identified the exact TSS using RACE. A cDNA fragment of approximately 350 bp was cloned and sequenced, which showed that *pri-mir-142* started at −1,205 within the upstream CGI (Figure 2B). Our data were not in agreement with the previously reported TSS at −308, identified using RACE with RNA from K562-derived cells [40]. To address this, we performed RACE also with RNA from the K562. We also included the PBPCs as they actively transcribe *mir-142*. However, we mapped the TSS to −1,205 also in these cells, indicating that mesenchymal and hematopoietic cells utilize the same promoter region.

Furthermore, we identified 2 polyadenylation signals using the Poly(A) Signal Miner [53], located 327–332 bp and 978–983 bp downstream of *pre-mir-142* (Figure 2B). Using RACE, the proximal poly(A) site was determined to be located 12 bp downstream of the first poly(A) signal in both MG-63, K562 and PBPCs, which indicated that *pri-mir-142* had a length of 1,636 nt in all cell types (Figure 2C). We performed an additional RACE to determine if the distal signal was functional. A corresponding amplicon of approximately 600 bp could only be detected in the PBPCs. This terminated 1,411 bp downstream of *pre-mir-142*, suggesting that this variant of *pri-mir-142* had a length of 2,703 nt (Figure 2C and S2 for sequences).

### *pre-mir-142* upstream region has promoter activity in mesenchymal cells when unmethylated

**Figure 3:**
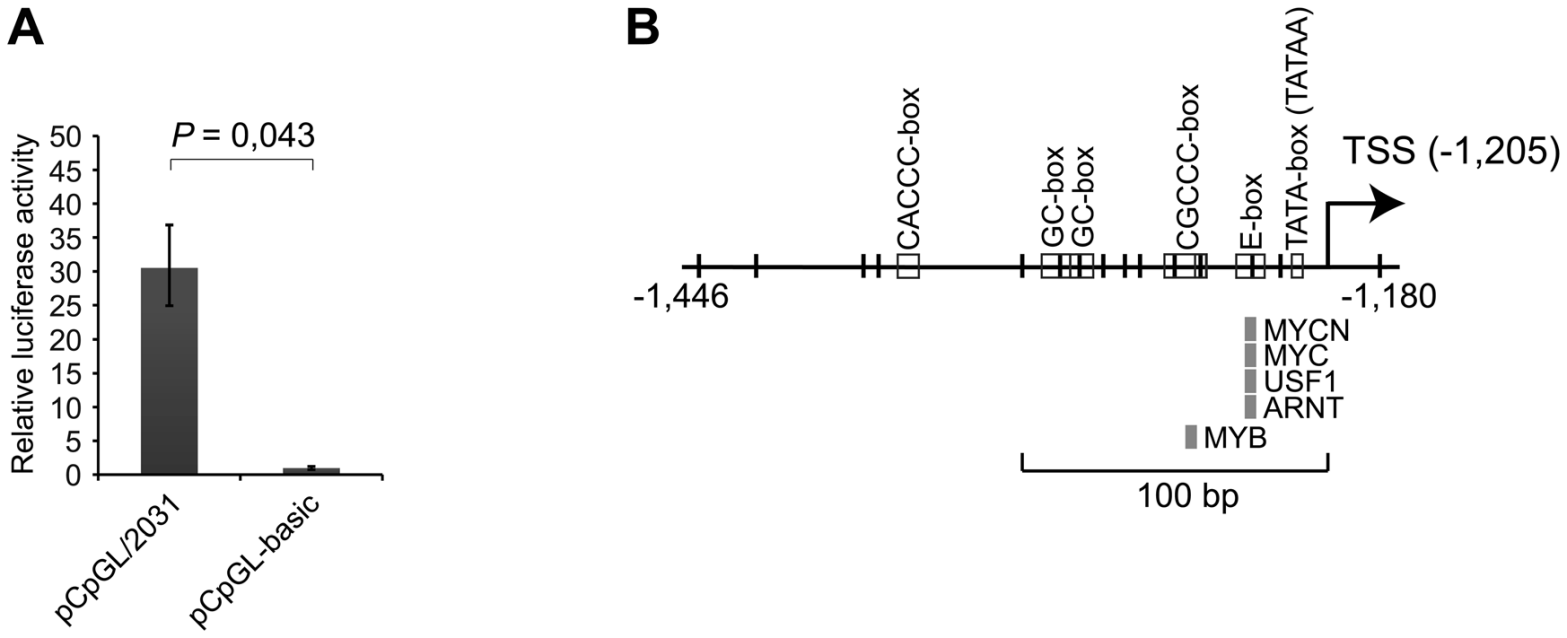
Assessment of the promoter activity of the upstream region of precursor *mir-142* and identification of the putative core/proximal *mir-142* promoter. (**A**) The 2,031 bp upstream region of *pre-mir-142* was cloned into the promoter-less luciferase reporter construct pCpGL-basic to assess its promoter activity. The resulting pCpGL/2031 construct and empty pCpGL-basic were transfected into U-2 OS cells along with a *Renilla* reporter construct. The luciferase activity was measured after 48 hours and calculated relative to that of pCpGL-basic (set to 1). Each histogram shows the average relative luciferase activity, and the error bars show the standard deviation of biological experiments (n≥5). The Wilcoxon signed rank test was used to test for statistical differences. A *P* value ≤0.05 was considered as significant. (**B**) Schematic representation of the putative core/proximal *mir-142* promoter region. The positions of the TATA-box, an enhancer box (E-box), 2 GC-boxes, a CACCC-box and a CGCCC-box (open boxes), and conserved binding sites for several transcription factors (filled boxes) are shown. Each vertical line represents an individual CpG. The numbers show the position relative to precursor *mir-142* (+1 to +87). TSS, transcription start site.

The above findings suggested that transcription of *mir-142* is blocked by DNA methylation of regulatory elements in cells with low levels of miR-142-5p/3p. To address this, the 2,031 bp region upstream of *pre-mir-142* was cloned into the CpG-deficient luciferase reporter construct pCpGL-basic. The resulting pCpGL/2031 construct and the empty pCpGL-basic were transfected into mesenchymal U-2 OS cells and luciferase activities were measured 48 hours after transfection. In cells transfected with pCpGL/2031, a significant approximately 30-fold increase of luciferase activity compared with control cells was observed (*P* = 0.043, Wilcoxon signed rank test) (Figure 3A). This indicated that the upstream region had intrinsic promoter activity in mesenchymal cells and that the required TFs to promote transcription of *mir-142* were present.

### Identification of the putative *mir-142* promoter

The colocalization of the TSS and upstream CGI suggested that this region might contain regulatory elements important for transcription of *mir-142*. To investigate this, we used the UCSC Genome Browser to search for conserved binding sites for TFs in the 2 kb upstream region of *pre-mir-142*. Interestingly, of the 9 TF-binding sites identified, 5 (v-myc myelocytomatosis viral related oncogene, neuroblastoma derived (MYCN); v-myc myelocytomatosis viral oncogene homolog (MYC); upstream transcription factor 1 (USF1); aryl hydrocarbon receptor nuclear translocator (ARNT); v-myb myeloblastosis viral oncogene homolog (MYB)) were located within 100 bp upstream of the TSS in a highly conserved approximately 265 bp region that encompassed both the TSS and CGI (Figure 3B). All TF-binding sites except that for MYB were associated with an E-box. In addition, we identified a TATA-box and several promoter-proximal elements, including a CGCCC-box and 2 GC-boxes (Figure 3B). Next, we used the ENCODE ChIP-seq dataset to search for enrichment of TF-binding upstream of *pre-mir-142* (data not shown). Besides the aforementioned MYC and USF1, binding sites for activating transcription factor 3 (ATF3), upstream transcription factor 2 (USF2), MYC associated factor X [64], and early growth response 1 (EGR1), as well as TATA box binding protein (TBP) and TATA box binding protein-associated factor 1 (TAF1) were enriched in the conserved region. Thus, we believe that this region represents the core/proximal *mir-142* promoter.

### Methylation status of CGIs associated *mir-142* inversely correlate with expression of miR-142-5p/3p upon treatment with 5-Aza and between mesenchymal and hematopoietic cells

**Figure 4:**
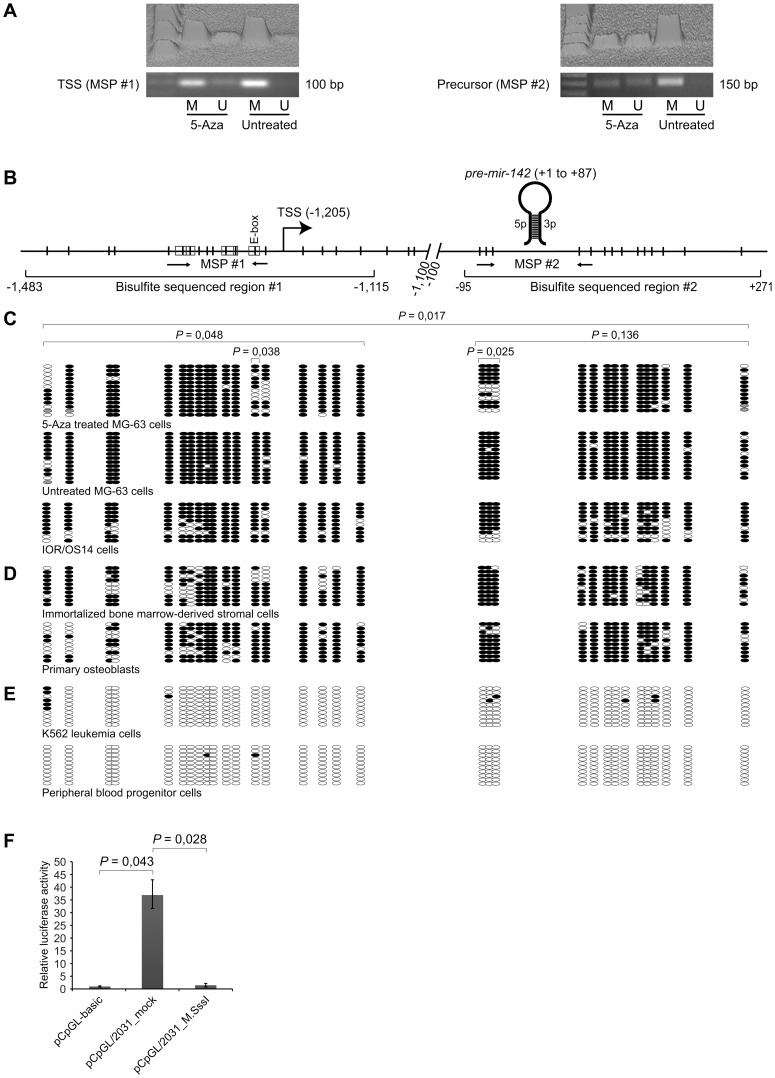
Analyses of methylation status of CGIs associated *mir-142* and *in vitro* methylation of its upstream regulatory region. (**A**) Methylation-specific PCR (MSP) analyses of the CGIs, before and after treatment with 5-Aza-2′-deoxycytidine (5-Aza). U and M, unmethylated and methylated products. Both standard gel images and 3D densitograms of the signal intensities are shown. (**B**) Schematic representation of the *mir-142* locus. Locations of MSP amplicon #1 and #2 are indicated by arrows. 18 CpGs (region #1) and 14 CpGs (region #2) were subjected to bisulfite sequencing. CpG-containing transcription factor binding motifs are enclosed by boxes (see Figure 3B for more information). E-box, enhancer box. Numbers indicate the position relative to *pre-miR-142* (+1 to +87). (**C**) Bisulfite sequencing of DNA from MG-63 cells before and after treatment with 5-Aza for 72 hours, and untreated IOR/OS14 cells. The Wilcoxon signed rank test was used to test for statistical differences between treated and untreated MG-63 cells, as described in more details in Materials and Methods. A *P* value ≤0.05 was considered as significant. (**D**) Bisulfite sequencing of immortalized bone marrow-derived stromal cells (iMSC#3) and primary osteoblasts. (**E**) Bisulfite sequencing of K562 leukemia- and peripheral blood progenitor cells. Black and white circles represent methylated and unmethylated CpGs, respectively, and each row represents a single clone. Grey circles, not determined. Ten clones were sequenced (n = 10), with the exception of MG-63 cells (region #1, n = 13; region #2, n = 12). (**F**) The 2,031 bp upstream region of *pre-mir-142* was cloned into the promoter-less luciferase reporter construct pCpGL-basic and *in vitro* methylated with M.SssI (pCpGL/2031_M.SssI) or mock-methylated (pCpGL/2031_mock). All three constructs were individually transfected into U-2 OS cells along with a *Renilla* reporter construct. The luciferase activity was measured after 48 hours and calculated relative to that of pCpGL-basic (set to 1). Each histogram shows the average relative luciferase activity, and the error bars show the standard deviation of biological experiments (n≥5). The Wilcoxon signed rank test was used to test for statistical differences and the *P* values are shown above the histograms. A *P* value ≤0.05 was considered as significant.

To further determine how DNA methylation controls *mir-142* transcription, we used MSP and bisulfite sequencing. First MSP was used to assess the methylation status of a region directly upstream of the TSS in mesenchymal MG-63 cancer cells, which have low endogenous levels of miR-142-5p/3p (Figure 1A), and found that it was hypermethylated (Figure 4A and S1 for control reactions). Next, we investigated if the increased expression of miR-142-5p/3p upon 5-Aza treatment (Figure 1B) was associated with reduced methylation. MSP is not a quantitative method, but the result suggested a decrease and an increase in methylated and unmethylated DNA, respectively (Figure 4A). In addition, we assessed the methylation status of the CGI encompassing *pre-mir-142*. This region also seemed to be heavily methylated and less so after 5-Aza treatment (Figure 4A).

To confirm these results and to obtain additional information regarding the methylation status of individual CpGs, we carried out bisulfite sequencing of 2 regions flanking the CpGs investigated by MSP. Region #1 from −1,483 to −1,115 covered the TSS and 18 CpGs, whereas region #2 from −95 to +271 encompassed *pre-mir-142* and 14 CpGs (Figure 4B). The bisulfite sequencing confirmed the MSP data and showed that treatment with 5-Aza resulted in a significant reduction (Wilcoxon signed rank test) of methylation of the upstream region #1 (*P* = 0.048), region #1 and #2 combined (*P* = 0.017), but not region #2 alone (*P* = 0.136). Furthermore, the CpG found in the E-box upstream of the TSS in region #1, and the 3 CpGs directly upstream of *pre-mir-142* in region #2, were significantly demethylated (*P* = 0.038 and 0.025, respectively) (Figure 4B and C). As examined by bisulfite sequencing, both CGIs were also heavily methylated in another osteosarcoma cell line (IOR/OS14), which further supported that expression of *mir-142* is directly or indirectly repressed by DNA methylation (Figure 4C). As the above studies were performed using tumorigenic cells we bisulfite sequenced DNA from normal mesenchymal cells (iMSC#3 and primary osteoblasts), which also have low levels of miR-142-5p/3p. Although less pronounced, both CGIs were also heavily methylated in these cells (Figure 4D). Accordingly, DNA methylation of the *mir-142* locus appeared to be a general feature of mesenchymal cells.

The above findings and the identification of the same TSS in representatives of mesenchymal and hematopoietic cells suggested that transcription of *mir-142* could be regulated by similar mechanisms in both cell types. However, we expected that the CGIs would be less methylated in the latter cells as they expressed miR-142-5p/3p at high levels (Figure 1A). To investigate this, we performed the bisulfite sequencing analyses with DNA from K562 cells and PBPCs, and found both CGIs to be almost completely unmethylated (Figure 4E). Thus, the methylation level of both CGIs inversely correlated with *mir-142* expression in mesenchymal and hematopoietic cells.

### Transcription of *mir-142* driven by the upstream region is directly silenced by *in vitro* DNA methylation

The observed partial demethylation of the CGIs and concomitant increase of *pri-mir-142* and mature miR-142-5p/3p after treatment with 5-Aza indicated that expression was regulated by DNA methylation. To provide evidence for repression of *mir-142* transcription being caused by methylation of an upstream regulatory region and not some indirect effects of 5-Aza, the 2,031 bp upstream region previously cloned into the promoter-less CpG-deficient luciferase reporter construct pCpGL/2031, was *in vitro* methylated using the methyltransferase M.SssI. A mock-methylated version of the same construct was used as control (pCpG/2031_mock). Transfection of U-2 OS cells with mock-treated pCpGL/2031 resulted in a significant approximately 35-fold increase of luciferase activity (*P* = 0.043, Wilcoxon signed rank test), compared with cells transfected with the empty pCpGL-basic (Figure 4F). Most importantly, when *in vitro* DNA methylated, the luciferase activity of pCpGL/2031 was significantly reduced to the basal level (*P* = 0.028, Wilcoxon signed rank test), indicating that transcription was directly repressed by DNA methylation (Figure 4F).

### Ectopic expression of miR-142-5p/3p impairs proliferation and might affect colony formation and migration

To investigate the basic functional consequences of epigenetic silencing of *mir-142* in mesenchymal cells, we first transfected miR-142-5p/3p mimics into MG-63 cells (Figure S3A), and obtained results that suggested an inhibition of cell proliferation, compared with cells transfected with a negative control mimic. As miR-142-5p/3p have different seed sequences, we also transfected the cells with individual mimics, thereby showing that only 3p had a significant effect on proliferation after 48 hours and until the experiment was ended (*P* = 0.043, Wilcoxon signed rank test). Similar results were obtained using the mesenchymal-like MDA-MB-231 breast cancer cells (data not shown). Furthermore, we evaluated the effects of stable overexpression of miR-142-5p/3p in MG-63 cells on proliferation, migration and colony formation (Figure S3B, C and D). Consistent with the results from the transient experiments, the cells appeared to proliferate slower than control cells transfected with an empty construct, although not significantly. In addition, ectopic expression of miR-142-5p/3p might stimulate migration and reduce the colony forming abilities of the cells. However, similarly as the effect on proliferation, these effects were not significant.

### The antisense *mir-142* strand is expressed in brain cells but not in mesenchymal and hematopoietic cells

**Figure 5:**
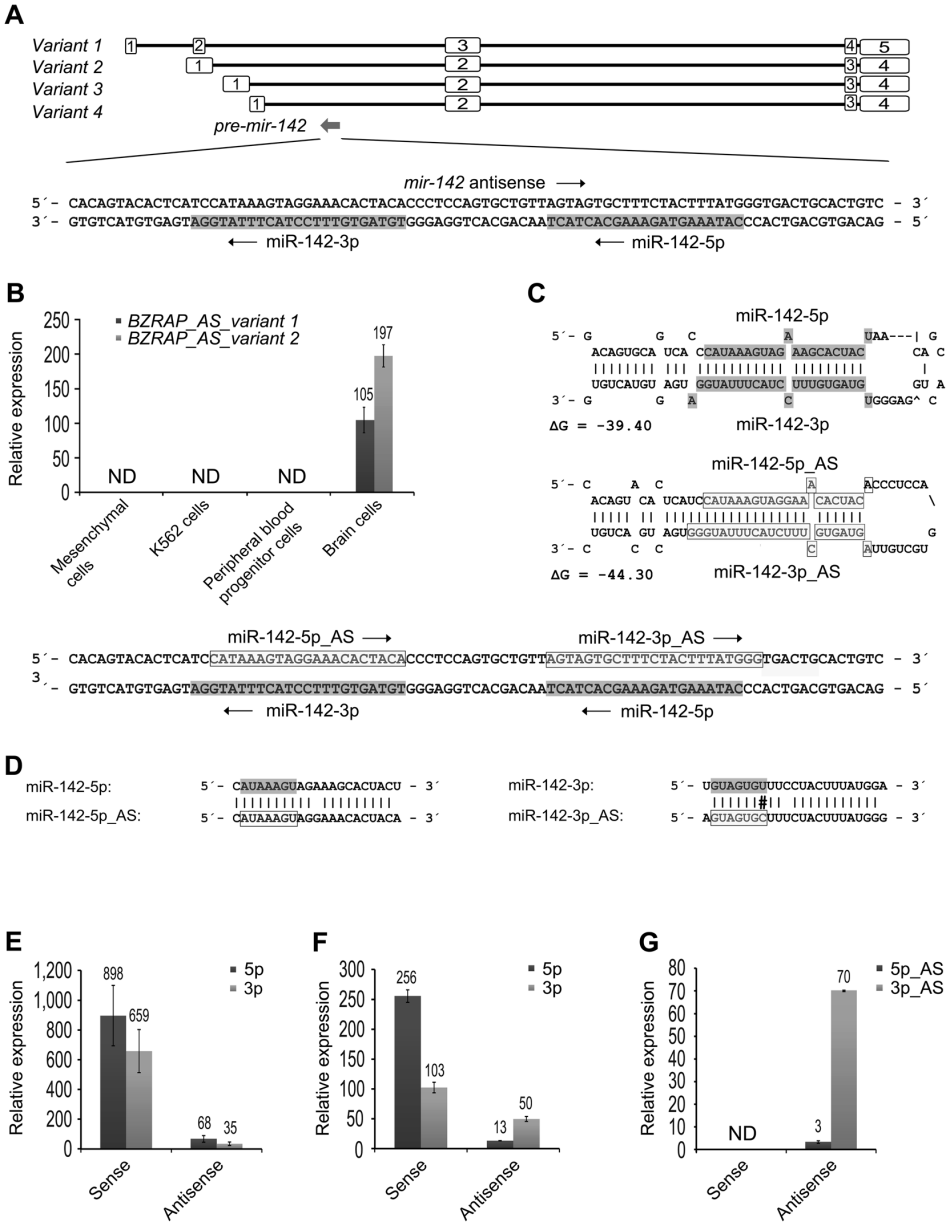
*In silico* and functional characterization of *mir-142* antisense transcripts. (**A**) Schematic representation of the *mir-142* locus. The location and transcriptional direction of *pre-mir-142* (gray arrow), relative to different transcript variants of *BZRAP1-AS1* are shown. Open boxes and black lines represent exons and introns, respectively. The locations of the mature sense miR-142-5p/3p in the precursor sequence are indicated by dark grey boxes. Arrows show the direction of transcription. (**B**) Expression levels of 2 transcript variants of *BZRAP1-AS1* in mesenchymal cells (MG-63, OHS, IOR/OS14 and iMSC#3), K562 cells, peripheral blood progenitor cells and brain cells as determined by qPCR. The Universal ProbeLibrary Assays were designed to cover the intron with antisense *mir-142*. As *BZRAP1-AS1* was only detected in brains cells, the expression levels were calculated relative to the expression of primary *mir-142* in the MG-63 cells (set to 1). Glyceraldehyde 3-phosphate dehydrogenase (*GAPDH*) was used for normalization. ND, not detected. (**C**) Secondary stem-loop structures of sense and antisense strands of *pre-mir-142* (predicted using Mfold). ΔG, minimum free energy; AS, antisense. (**D**) Alignment of mature sense and predicted antisense sequences. Lines depict perfect Watson-Crick pairing. #, mismatch in seed region. (**E–G**) Cells with low endogenous levels of miR-142-5p/3p were transiently (OHS cells in E), or stably (MG-63 cells in F and G) transfected with expression constructs with sense and antisense strands of *pre-mir-142*. The levels of mature miRNAs were determined using qPCR with either standard (E and F) or customized (G) antisense TaqMan assays. Numbers above the histograms show the expression levels, relative to cells transfected with an empty construct (set to 1). As the antisense assays gave no signal in the control cells, the C_T_-value was arbitrarily set to 38. 5p and 3p represent miR-142-5p and 3p, respectively. AS, antisense miR-142. *RNU44* was used for normalization. ND, not detected. The error bars in all qPCR experiments show the standard deviation of technical replicates.

During the characterization of the *miR-142* locus we discovered that *mir-142* was located in an intron of the non-coding gene *BZRAP1-AS1*, but in the antisense orientation (Figure 5A). *BZRAP1-AS1* encodes 9 transcript variants, and cDNAs corresponding to number 1 and 2 have been cloned from brain tissue [65]. Interestingly, the first transcript variant was associated with a large CGI, which as assessed by MSP was hypermethylated in mesenchymal cells (data not shown). To investigate if *BZRAP1-AS1* was involved in the regulation of *mir-142*, we used qPCR assays designed to span the boundaries of the *mir-142*-containing introns of transcript variant 1 and 2 (Table S1). We did not detect any signals in mesenchymal cells (MG-63, OHS, IOR/OS14, iMSC#3) neither before nor after treatment with 5-Aza, or in hematopoietic cells (K562 and PBPCs), although low expression of both transcripts was detected in the brain positive control RNA (Figure 5B).

### The antisense *pre-mir-142* strand is predicted to adopt a miRNA precursor hairpin structure and might give rise to mature miRNAs

Interestingly, the antisense strand of the *mir-142* precursor sequence was predicted to adopt a hairpin structure typical for miRNA precursors, based on the structural parameters and minimal free energy (Figure 5C). Moreover, *pre-miR-142* is highly conserved in vertebrates (Figure S4) and a sequence comparison of the mature miR-142-5p/3p and their predicted antisense counterparts, showed that they were very similar (Figure 5D). Thus, we reasoned that the qPCR assays designed to detect the mature sense miRNAs might also recognize the antisense versions (although most likely with a reduced efficiency). To investigate this, we cloned *pre-mir-142* with flanking sequences in either the sense or antisense orientation. Then we transfected constructs with both orientations into OHS cells. The sense *mir-142* strand was efficiently processed into mature miRNAs, giving relative increases of approximately 600–900-fold, but the antisense construct also gave approximately 30–70-fold increases of miR-142-5p/3p as detected by the standard qPCR assays (Figure 5E). We also generated an MG-63-derived cell line with stable expression of *mir-142* antisense and observed approximately 13–50-fold increases of miR-142-5p/3p, compared to approximately 100–260-fold increases for the sense construct (Figure 5F). To further study this, we designed customized TaqMan assays to specifically detect the antisense transcripts. These assays only gave signals in cells with ectopic expression of the *mir-142* antisense strand, which suggested that they were highly specific for miR-142-5p/3p antisense (Figure 5G). However, using these assays the antisense miRNAs were not detected in brain cells although their host gene *BZRAP1-AS1* was expressed at low levels (data not shown). As we already had shown that ectopic expression of miR-142-5p/3p might affect the phenotype of MG-63 cells (Figure S3), we wanted to investigate if stable expression of miR-142-5p/3p antisense could function in a similar manner. However, the observed effects on proliferation, migration and colony forming ability were not significant based on two biological experiments (Figure S3B, C and D).

## Discussion

Epigenetic mechanisms such as DNA methylation and covalent modifications of histones seem to play a crucial role in controlling miRNA gene expression [25]. However, the mechanisms underlying the tissue- and cell type-specific regulation of miRNAs have in most cases not been extensively characterized. The mature miRNAs derived from the conserved *mir-142* locus are preferentially expressed in hematopoietic cells [19], [22], [23], [33], and at variable but low levels in various cells of mesenchymal origin. To investigate the involvement of epigenetic silencing, we first treated mesenchymal cells with the demethylating agent 5-Aza, and showed that this resulted in a concomitant increase of *pri-mir-142* and mature miR-142 transcripts. DNA methylation of CGIs is known to be associated with repression of transcription [30], and the observation that approximately 50% of human miRNA genes have CGIs [29] suggests that they are regulated by epigenetic mechanisms. Accordingly, a large number of epigenetically repressed miRNAs has been discovered (reviewed in [5]). An upstream CGI and a CGI embedding the precursor sequence were identified for *mir-142*. The former CGI is especially interesting as we found it to be colocalized with the TSS of *mir-142* located 1,205 bp upstream of the precursor in all cell types investigated here, thus suggesting that *mir-142* might be regulated by DNA methylation of a promoter-associated CGI.

To provide further evidence to support this, we used MSP and bisulfite sequencing to demonstrate that both CGIs are hypermethylated in normal and tumorigenic mesenchymal cells. In addition to induced miRNA expression, treatment with 5-Aza also resulted in a weak, but significant reduction in methylation of the CGI overlapping the TSS, which shows that there is an inverse correlation between methylation of this region and transcription of *mir-142*. Furthermore, we used a CpG-free luciferase reporter construct to show that *in vitro* methylation of the upstream region with the promoter-associated CGI significantly repressed its transcriptional activity. Together, our data indicate that the required transcriptional machinery to promote transcription of *mir-142* is present in mesenchymal cells, but that methylation prevents transcription. The regions located immediately upstream of genes frequently exhibit high levels of sequence conservation due to the presence of critical *cis*-regulatory elements [66]–[68]. In the case of *mir-142*, a highly conserved approximately 265 bp region overlapped with the upstream CGI and TSS, which by the ENCODE ChIP-seq data was shown to be occupied by TBP and TAF1. Although relatively few miRNA TF-binding sites have been experimentally verified, their distribution has been shown to overlap with those of predicted CGIs and TSSs [69]. In agreement with this, we observed an enrichment of conserved binding sequences for USF1, MYC, MYB, MYCN, and ARNT. An experimental verification of these was beyond the scope of this study, but the ENCODE ChIP-seq data confirm binding of the 2 former TFs. These TFs are transcribed also in mesenchymal cells ([62], GEO accession number GSE28425) and could promote transcription of *mir-142* also here. Interestingly, USF1, MYC, MYCN and ARNT were associated with an E-box, which is a positive regulator of transcription. Methylation of E-boxes, as observed in the present study, has been shown to significantly decrease their binding affinity for TFs, such as the aforementioned USF1 [70] and MYC [71]. Thus, direct inhibition of TF-binding by DNA methylation might also be an important mechanism in the epigenetic regulation of *mir-142*, as bisulfite sequencing revealed that the methylation of the CpG associated with the E-box was significantly reduced after 5-Aza treatment. Together, the above data provide compelling evidence that the conserved approximately 265 bp region represents the *mir-142* core/proximal promoter. In support of this, the CoreBoost_HM promoter prediction algorithm, which is based on DNA sequence features and epigenetic information, predicts the core promoter and TSS of *mir-142* to be located precisely there [8].

Increasing evidence support an important role of epigenetic mechanisms in regulation of cell type-specific miRNA expression [25], [72]. Intriguingly, both the upstream and precursor-embedding CGIs were found to be unmethylated in K562 and PBPCs where miR-142-5p/3p are constitutively expressed. This raised the possibility that DNA methylation *per se* could be an important factor in repressing transcription of *mir-142* in non-hematopoietic cells [19], [73]. Consistent with this notion, miR-142-5p/3p increase in colon cancer cells after treatment with 5-Aza [74]. Furthermore, according to the ENCODE/HAIB genome-wide methylation data representing a large variety of cell types, the *mir-142* locus only appears to be unmethylated in cells of hematopoietic origin, while it is heavily methylated in all other investigated tissues.

As we focused mainly on DNA methylation, the presence of relevant chromatin marks at the *mir-142* locus was not experimentally determined. However, based on ENCODE/Broad ChIP-seq data the high expression of miR-142-5p/3p in K562 cells was associated with permissive histone marks, e.g. H3K27ac, H3K79me2 and H3K4me3, whereas repressive marks including H3K9me3 and H3K27me3 were absent. On the other hand, consistent with other miRNAs whose repression was associated with DNA methylation, the permissive marks were absent in osteoblasts with low expression of miR-142-5p/3p [25], [75]. Moreover, the repressive marks were also absent. In a recent study by Vrba *et al* [25] tissue-specific miRNAs were in most cases repressed by either DNA methylation (38%) or H3K27me3 (58%), whereas only a small set of miRNAs were regulated by both epigenetic modifiers (21%). Accordingly, the absence of H3K27me3, as well as H3K9me3, strengthens the role of DNA methylation as a major repressor of *mir-142* transcription in mesenchymal cells. Furthermore, acetylation of histones is positively correlated with expression (reviewed in [32]). In agreement with this, H3K27ac was enriched and absent at the *mir-142* locus in K562 cells and osteoblasts, respectively. For some miRNAs, treatment with 5-Aza combined with a histone deacetylase inhibitor may have an additive effect on their induction, compared with individual treatment with either 5-Aza or the histone deacetylase inhibitor [63], [76]. However, this was not observed for either primary or mature *mir-142* transcripts in mesenchymal MG-63 cells, suggesting that histone deacetylation is not a major contributor to the repression of *mir-142* in these cells.

In systemic lupus erythematosus CD4 positive T cells, downregulation of miR-142-5p/3p correlates with increased methylation of the 3 CpGs directly upstream of *pre-mir-142* and an enrichment of H3K27me3 [40]. Thus, this indicates that epigenetic events might be involved in regulation of *mir-142* also in cells of hematopoietic origin. This study performed their experiments based on the assumption that the TSS is located at −308, which was reported in a previous study [40]. Consequently, the promoter-associated CGI that we identified was not analyzed.

The enrichment of miR-142-5p/3p in hematopoietic cells reflects their involvement in regulating immune cell functions [19], [34]–[37], [39], [77]. In mesenchymal cells, methylation and repression of *mir-142* could allow for high expression of genes consistent with a mesenchymal phenotype. Two such candidates could be the predicted targets zinc finger E-box binding homeobox 1 and 2 (ZEB1/2) [78], which are expressed in mesenchymal cells (data not shown). Moreover, we found a significant inhibition of proliferation in mesenchymal MG-63 cells only by miR-142-3p and also observed that ectopic expression of miR-142-5p/3p might stimulate migration and repress the colony forming ability of the same cells. Similarly, overexpression of miR-142-3p has been shown to inhibit proliferation and colony forming ability of primitive hematopoietic cells [79]. Some of these functions appear to depend on the cellular type and context, as miR-142-3p has been reported to act as an anti-migratory factor in hepatocellular carcinoma cells [80]. Nevertheless, the finding that *mir-142* possibly can affect the cellular phenotype of mesenchymal cells is interesting, as miR-142-5p/3p have been reported to be deregulated in mesenchymal tumors [81], [82]

Several studies have looked at transcriptional features associated with miRNA genes (reviewed in [83]). However, the structure of most miRNA genes, including that of *mir-142*, has not previously been experimentally determined. We showed that the TSS and proximal polyadenylation site of *mir-142* in mesenchymal and hematopoietic cells are located at −1,205 and +431, respectively. Accordingly, we propose that *pri-mir-142* has a length of 1,636 nt, a finding also supported by expressed sequence tags, data from high-throughput sequencing and a previously cloned 1,642 nt transcript (GenBank: AX721088). In addition, we identified a distal, less conserved polyadenylation site utilized in the PBPCs. This finding warrants further investigations, as we did not find any evidence in the databases to support its existence. However, polyadenylation sites frequently vary across cell types [84].

The generation of sense and antisense miRNAs from the same locus was first described in *Drosophila* [9]–[11], and an increasing number of low abundance mature miRNAs are now being mapped to the antisense strand of previously annotated loci [12], [13], [85]. Several lines of evidence indicate that functional mature miRNAs might also derive from the antisense strand of *mir-142* under given circumstances. First, the minimal free energy structure of the predicted precursor is a characteristic miRNA hairpin with 2 nt 3′- overhangs. The antisense miR-142-5p/3p are very similar to their sense counterparts, the most noteworthy difference being a cytosine instead of uracil in the seed sequence of antisense miR-142-3p, which suggests distinct targeting. Moreover, upon ectopic expression we could specifically detect both of the antisense mature transcripts by customized qPCR assays, and the antisense miRNAs might inhibit proliferation in a similar manner as did the sense miRNAs. Interestingly, there might also be functional differences between sense and antisense, as we observed no effect on migratory properties when overexpressing the antisense strand. However, as none of these experiments exhibited statistical significance, these results clearly warrant further investigations. In addition, we were not able to detect the mature antisense miRNAs, neither in brain, where their putative host gene is expressed at low levels, or in mesenchymal cells. This could be attributed to the fact that most known antisense miRNAs are very low in abundance with strict developmental, cell-type and stage-specific expression patterns [13], [85]. Thus, the relevance of antisense miR-142-5p/3p remains to be further elucidated.

In summary, we have demonstrated that *mir-142* is epigenetically regulated in mesenchymal cells. We believe that transcription of the 1,636 nt primary transcript is repressed by extensive DNA methylation of an upstream promoter-associated CGI. The repression could be due to inhibition of TF-binding, as several CpG-containing regulatory elements were identified in the vicinity of the TSS. DNA methylation *per se* could also be involved in repressing *mir-142* in various other cell types, as the *mir-142* locus was found to be unmethylated in hematopoietic cells. Furthermore, consistent with other reports, miR-142-5p/3p are involved in cellular behavior as they can modulate the proliferation, and possibly also migration and colony forming ability of mesenchymal cells. Finally, the discovery that ectopic expression of the antisense strand of *pre-miR-142* might give rise to functional miRNAs reinforces the recent concept of antisense transcription of annotated human miRNA genes. Our study is the first to elucidate mechanisms responsible for repression of *mir-142* in mesenchymal cells and expands our knowledge about the regulation and function of miR-142-5p/3p. Further characterization of the *mir-142* locus and other miRNAs will be of great importance for a better understanding of the diverse roles of this class of ncRNAs during normal development and differentiation, as well as in human disease.

## Supporting Information

Figure S1 **Control reactions for methylation-specific PCR.** (**A**) Control reactions for primers designed to assess the methylation level of CpGs located approximately 1,300 bp upstream of the precursor sequence. (**B**) Control reactions for primers designed to assess CpGs in the immediate flanking regions of the precursor sequence. Methylated and unmethylated bisulfite converted DNA, plus unconverted DNA were used for optimization and control experiments. Water was used as a negative control. U and M, unmethylated and methylated products.
(TIF)

Figure S2 **Sequences of primary** ***mir-142*** **transcripts.** Two transcript variants of *mir-142* were identified using rapid amplification of cDNA ends (1,636 and 2,703 nucleotides). Sequences enclosed by yellow and red boxes depict the proximal and distal polyadenylation signals, respectively. Grey boxes indicate the location of precursor *mir-142* (87 nucleotides).
(TIF)

Figure S3 **Proliferation, migration and colony forming abilities of MG-63 cells transfected with miR-142-5p/3p mimics and** ***mir-142*** **sense or antisense strand.** Statistical significance was tested using a Wilcoxon signed rank test. A *P* value ≤0.05 was considered as significant (indicated by an asterisk above the histograms). (**A**) MG-63 cells were transfected with various combinations of synthetic miR-142-5p/3p mimics, and a negative control mimic. Cellular proliferation rates were determined by live cell imaging using the IncuCyte (Essens Bioscience). Error bars represent the standard deviation of biological experiments (n = 4). (**B**) Stable transfection of MG-63 cells with constructs expressing miR-142-5p/3p sense, antisense or an empty control construct (sense, antisense or control, respectively). Cellular proliferation rates were determined by live cell imaging using the IncuCyte. Error bars represent the standard deviation of biological experiments (n = 2). (**C**) Migration assay performed with the same cells as in B. Error bars represent the standard deviation of biological experiments (n = 2). (**D**) Colony forming assay performed with the same cells as in B. Error bars represent the standard deviation of biological experiments (n = 2).
(TIF)

Figure S4 **Evolutionary conservation of precursor** ***mir-142*** **.** Multiple alignment and PhastCons vertebrate conservation (46 species) of precursor *mir-142* (87 bp) using the UCSC Genome Brower. The precursor including the hairpin loop is highly evolutionary conserved in vertebrates. Grey boxes show the nucleotides that correspond to the sense and putative miR-142-5p/3p antisense miRNAs. The horizontal grey bar below the sense and antisense precursor sequences shows the PhastCons vertebrate conservation. Bold letters indicate unconserved nucleotides. AS, antisense.
(TIF)

Table S1 **Overview of primers used to clone the** ***mir-142*** **gene and Universal ProbeLibrary assays.** Tm, primer annealing temperature; TSS, transcription start site; Probe #, Universal ProbeLibrary probe number; *BZRAP1-AS1*, *BZRAP1* antisense RNA 1; ^1^, position of amplicon relative to the 5′- end of *mir-142* precursor sequence.
(DOC)

Table S2 **Overview of TaqMan expression assays used for quantitative real-time PCR.** hsa, *Homo sapiens*; *RNU44* (*SNORD44*), small nucleolar RNA, C/D box 44; *GAPDH*, glyceraldehyde-3-phosphate dehydrogenase; *MEST*, mesoderm specific transcript; ^#^, sequences designed based on predicted antisense sequences of miR-142-5p and 3p (Figure 5C); AS, antisense.
(DOC)

Table S3 **Overview of gene-specific primers used for rapid amplification of cDNA ends.** Tm, primer annealing temperature; GSP, gene-specific primer; ^1^, position of primer relative to the 5′- end of *mir-142* precursor sequence.
(DOC)

Table S4 **Overview of primers used for methylation-specific PCR and bisulfite sequencing.** Tm, annealing temperature; U and M, primers specific for unmethylated and methylated sequences; TSS, transcription start site; MSP; methylation-specific PCR; Pre, precursor sequence; ^1^, position of amplicon relative to the 5′- end of *mir-142* precursor sequence.
(DOC)

Table S5 **Overview of primers used to clone the putative regulatory region of the** ***mir-142*** **gene.** Tm, annealing temperature; ^1^, position of amplicon relative to the 5′- end of *mir-142* precursor sequence.
(DOC)
