# Supplementary figures and images for: Epigenetic Regulation and Functional Characterization of MicroRNA-142 in Mesenchymal Cells
Source: PLoS One. 2013 Nov 13;8(11):e79231. doi: 10.1371/journal.pone.0079231 (PMC3827369; doi:10.1371/journal.pone.0079231)

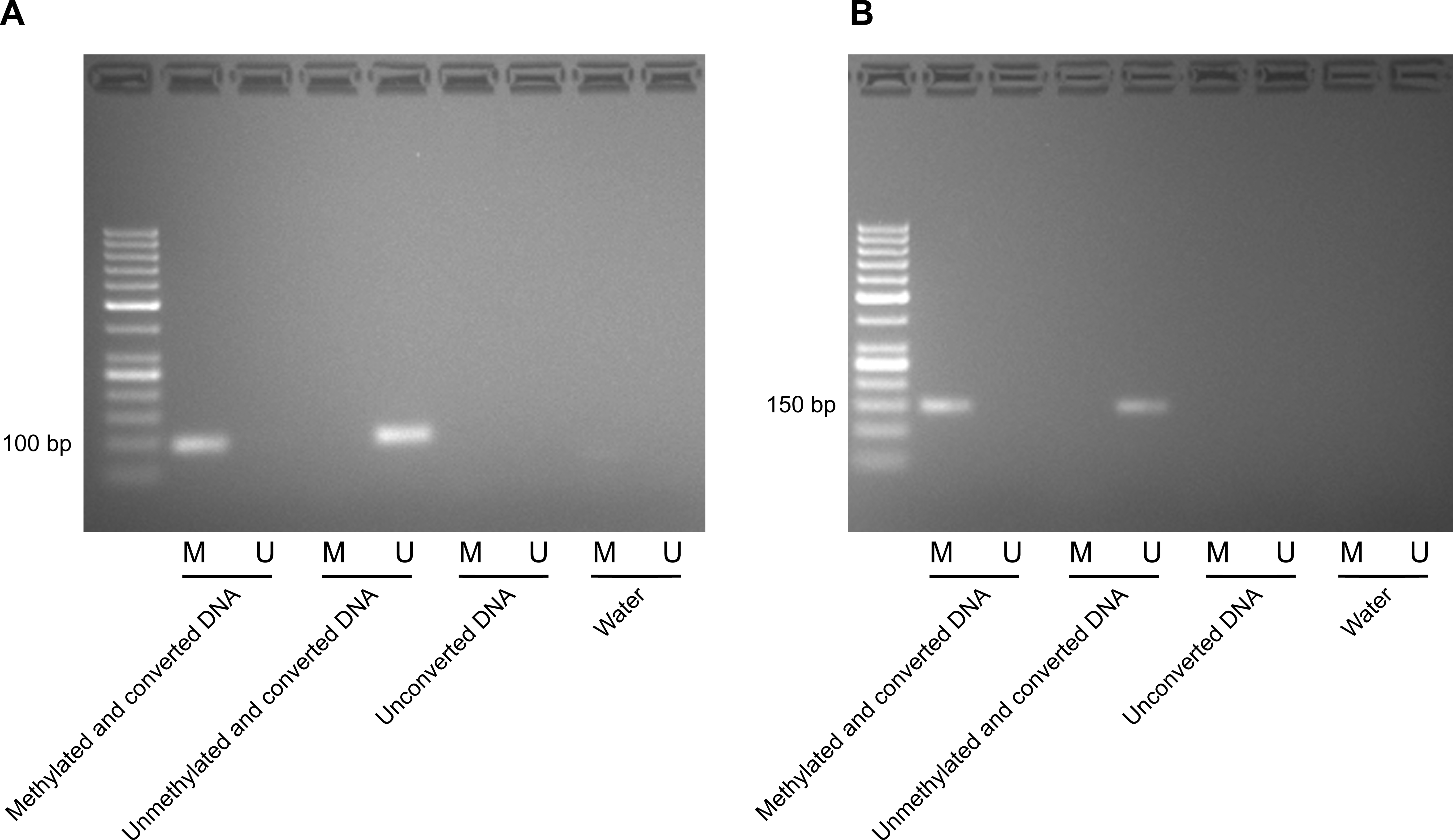

Supplement: Figure S1 — Control reactions for methylation-specific PCR. (A) Control reactions for primers designed to assess the methylation level of CpGs located approximately 1,300 bp upstream of the precursor sequence. (B) Control reactions for primers designed to assess CpGs in the immediate flanking regions of the precursor sequence. Methylated and unmethylated bisulfite converted DNA, plus unconverted DNA were used for optimization and control experiments. Water was used as a negative control. U and M, unmethylated and methylated products. (TIF) [file pone.0079231.s001.tif]

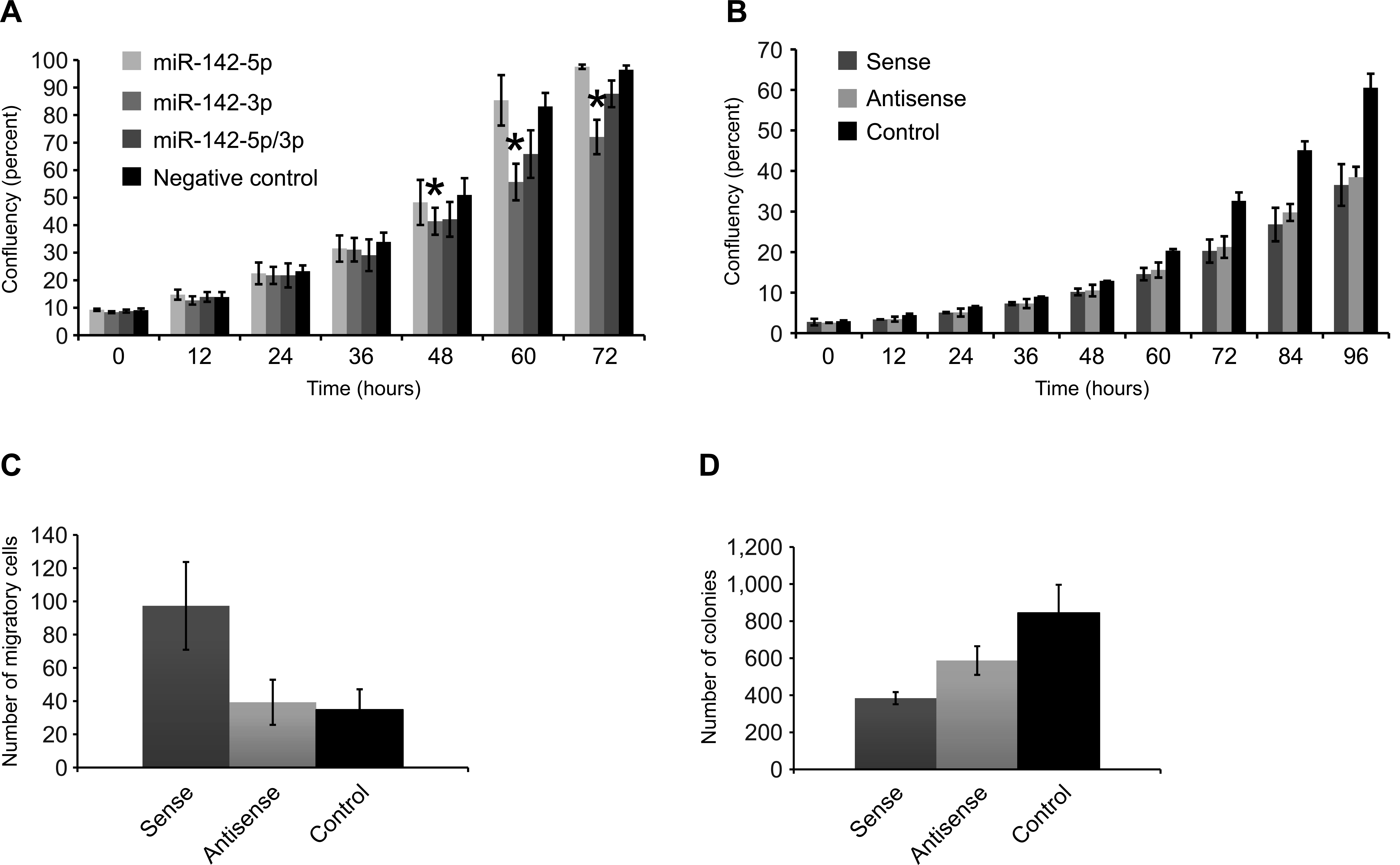

Supplement: Figure S3 — Proliferation, migration and colony forming abilities of MG-63 cells transfected with miR-142-5p/3p mimics and mir-142 sense or antisense strand. Statistical significance was tested using a Wilcoxon signed rank test. A P value ≤0.05 was considered as significant (indicated by an asterisk above the histograms). (A) MG-63 cells were transfected with various combinations of synthetic miR-142-5p/3p mimics, and a negative control mimic. Cellular proliferation rates were determined by live cell imaging using the IncuCyte (Essens Bioscience). Error bars represent the standard deviation of biological experiments (n = 4). (B) Stable transfection of MG-63 cells with constructs expressing miR-142-5p/3p sense, antisense or an empty control construct (sense, antisense or control, respectively). Cellular proliferation rates were determined by live cell imaging using the IncuCyte. Error bars represent the standard deviation of biological experiments (n = 2). (C) Migration assay performed with the same cells as in B. Error bars represent the standard deviation of biological experiments (n = 2). (D) Colony forming assay performed with the same cells as in B. Error bars represent the standard deviation of biological experiments (n = 2). (TIF) [file pone.0079231.s003.tif]

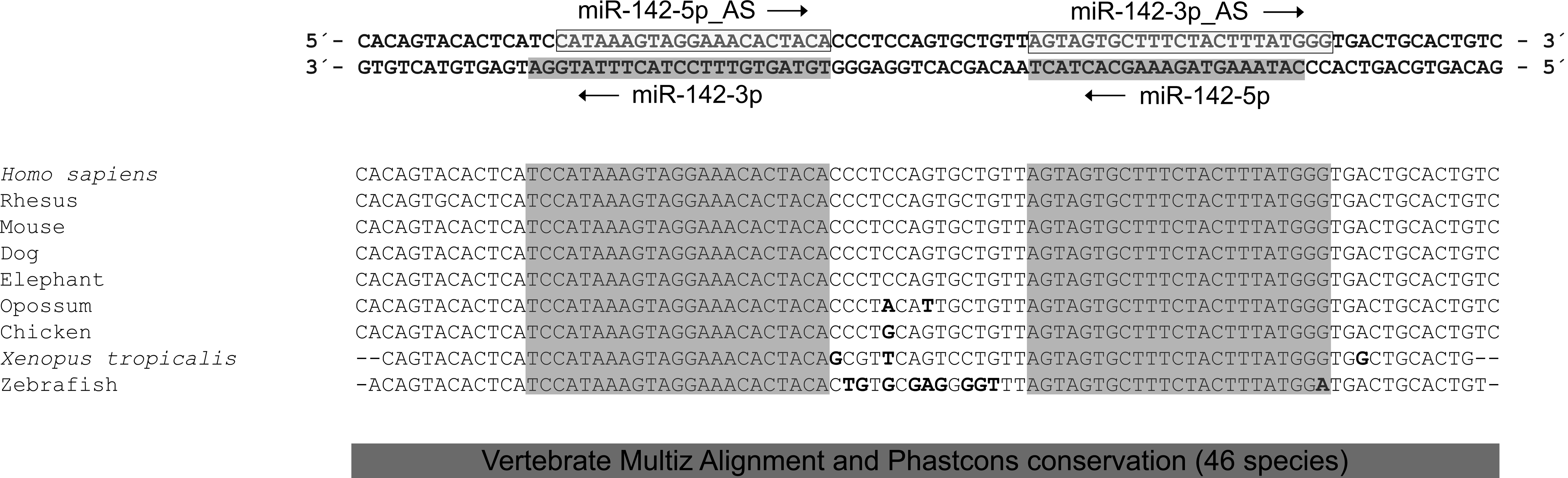

Supplement: Figure S4 — Evolutionary conservation of precursor mir-142 . Multiple alignment and PhastCons vertebrate conservation (46 species) of precursor mir-142 (87 bp) using the UCSC Genome Brower. The precursor including the hairpin loop is highly evolutionary conserved in vertebrates. Grey boxes show the nucleotides that correspond to the sense and putative miR-142-5p/3p antisense miRNAs. The horizontal grey bar below the sense and antisense precursor sequences shows the PhastCons vertebrate conservation. Bold letters indicate unconserved nucleotides. AS, antisense. (TIF) [file pone.0079231.s004.tif]
