# Supplementary material for: Epigenetic Regulation and Functional Characterization of MicroRNA-142 in Mesenchymal Cells
Source: PLoS One. 2013 Nov 13;8(11):e79231. doi: 10.1371/journal.pone.0079231 (PMC3827369; doi:10.1371/journal.pone.0079231)
Supplement: Table S1 — Overview of primers used to clone the mir-142 gene and Universal ProbeLibrary assays. Tm, primer annealing temperature; TSS, transcription start site; Probe #, Universal ProbeLibrary probe number; BZRAP1-AS1, BZRAP1 antisense RNA 1; 1, position of amplicon relative to the 5′- end of mir-142 precursor sequence. (DOC) [file pone.0079231.s005.doc]

**Table S1 Overview of primers used to clone the *mir-142* gene and Universal ProbeLibrary assays.**

| **Primer name** | **Forward sequence (5’- 3’)** | **Reverse sequence (5’- 3’)** | **Probe #** | **Tm (°C)** | **Size of amplicon (bp)** | **Positions^1^** |
| --- | --- | --- | --- | --- | --- | --- |
| *mir-142*_F_2 and *mir-142*_R_2 | GGAGTCAGGAGGCCTGGGCA | AGGGCAGCAGAGGAGCTGCT | - | 60 | 334 | -50 to +284 |
| **qPCR primers and probes for transcriptional characterization of *mir-142* locus** | | | | | | |
| TSS_*142*_qPCR_F_1 and R_1 | CCTTCCCTCGAGCCTCTTT | AGGTGCTATGCGGAGAGAAG | 25 | 60 | 60 | -1975 to -1914 |
| TSS_*142*_qPCR_F_2 and R_2 | CATGCATCAGCACCTCCAT | CCTTCCCTTCCCAACACTC | 81 | 60 | 78 | -1711 to -1632 |
| TSS_*142*_qPCR_F_3 and R_3 | ACTGGGGCTCCTCAGCTC | CACCCAAGCTCCTCGACA | 66 | 60 | 86 | -1122 to -1035 |
| TSS_*142*_qPCR_F_4 and R_4 | CACCATTTTTGTTGGGATAGC | CCAAAGTCCTGACTCCCTCA | 51 | 60 | 73 | -828 to -754 |
| **qPCR primers and probes for *BZRAP1-AS1* (transcript variant 1 and 2)** | | | | | | |
| BZRAP1-AS1_variant1_ex2-3_F and R | CACAAAGGAGGCTGCAGAA | GTCCTGGGATGTGAGGAATC | 81 | 60 | 87 | Spans the 9,325 bp intron with *mir-142* antisense |
| BZRAP1-AS1_variant 2_ex1-2_F and R | GTGCAGATGGTCCTCCCTAC | GTCCTGGGATGTGAGGAATC | 51 | 60 | 62 | Spans the 8,372 bp intron with *mir-142* antisense |

Tm, primer annealing temperature; TSS, transcription start site; Probe #, Universal ProbeLibrary probe number; BZRAP1-AS1, *BZRAP1* antisense RNA 1; ^1^, position of amplicon relative to the 5’- end of *mir-142* precursor sequence.
