# Supplementary material for: Epigenetic Regulation and Functional Characterization of MicroRNA-142 in Mesenchymal Cells
Source: PLoS One. 2013 Nov 13;8(11):e79231. doi: 10.1371/journal.pone.0079231 (PMC3827369; doi:10.1371/journal.pone.0079231)
Supplement: Table S2 — Overview of TaqMan expression assays used for quantitative real-time PCR. hsa, Homo sapiens; RNU44 (SNORD44), small nucleolar RNA, C/D box 44; GAPDH, glyceraldehyde-3-phosphate dehydrogenase; MEST, mesoderm specific transcript; #, sequences designed based on predicted antisense sequences of miR-142-5p and 3p (Figure 5C); AS, antisense. (DOC) [file pone.0079231.s006.doc]

**Table S2 Overview of TaqMan expression assays used for quantitative real-time PCR.**

| **Assay name** | **miRBase ID/"Official name or symbol"** | **Assay ID/Cat. No.** | **Assay type** |
| --- | --- | --- | --- |
| hsa-miR-142-5p | hsa-miR-142-5p | 002248 | Mature miRNA |
| hsa-miR-142-3p | hsa-miR-142-3p | 000464 | Mature miRNA |
| *RNU44* | *SNORD44*, small nucleolar RNA C/D box 44 | 001094 | Mature miRNA Control |
| *hsa-mir-142* | *hsa-mir-142* | Hs03303162_pri | Primary miRNA |
| *MEST* | *MEST* | Hs00853380_g1 | Messenger RNA |
| Human *GAPD* | *GAPDH* | Hs99999905_m1 | Endogenous control |
| **Overview of Custom TaqMan Small RNA Assays** | | | |
| **Assay name** | **Target sequence (5’- 3’)^#^** | **Assay ID** | **Assay type** |
| hsa-miR-142-5p_AS | CAUAAAGUAGGAAACACUACA | CSN1EJ8 | Mature miRNA |
| hsa-miR-142-3p_AS | AGUAGUGCUUUCUACUUUAUGGG | CSPACQG | Mature miRNA |

hsa, *Homo sapiens*; *RNU44* (*SNORD44*), small nucleolar RNA, C/D box 44; *GAPDH*, glyceraldehyde-3-phosphate dehydrogenase; *MEST*, mesoderm specific transcript; ^#^, sequences designed based on predicted antisense sequences of miR-142-5p and 3p (Figure 5C); AS, antisense.
