# Supplementary material for: Epigenetic Regulation and Functional Characterization of MicroRNA-142 in Mesenchymal Cells
Source: PLoS One. 2013 Nov 13;8(11):e79231. doi: 10.1371/journal.pone.0079231 (PMC3827369; doi:10.1371/journal.pone.0079231)
Supplement: Table S3 — Overview of gene-specific primers used for rapid amplification of cDNA ends. Tm, primer annealing temperature; GSP, gene-specific primer; 1, position of primer relative to the 5′- end of mir-142 precursor sequence. (DOC) [file pone.0079231.s007.doc]

**Table S3 Overview of gene-specific primers used for rapid amplification of cDNA ends**.

| **Primer name** | **Sequence (5’- 3’)** | **Tm (°C)** | **Positions^1^** |
| --- | --- | --- | --- |
| **5’- rapid amplification of cDNA ends** | | | |
| GSP_R3 | TCCCACTTCTCAGCCTCTGCGTACC | 68 | -897 to -872 |
| **3’- rapid amplification of cDNA ends** | | | |
| GSP_F2 | GGAGTCAGGAGGCCTGGGCA | 66 | -50 to -30 |
| GSP_F4 | GCAATGATAGAAACATAGGGCGTGT | 60/66 | +912 to +936 |

Tm, primer annealing temperature; GSP, gene-specific primer; ^1^, position of primer relative to the 5’- end of *mir-142* precursor sequence.
