# Supplementary material for: Epigenetic Regulation and Functional Characterization of MicroRNA-142 in Mesenchymal Cells
Source: PLoS One. 2013 Nov 13;8(11):e79231. doi: 10.1371/journal.pone.0079231 (PMC3827369; doi:10.1371/journal.pone.0079231)
Supplement: Table S4 — Overview of primers used for methylation-specific PCR and bisulfite sequencing. Tm, annealing temperature; U and M, primers specific for unmethylated and methylated sequences; TSS, transcription start site; MSP; methylation-specific PCR; Pre, precursor sequence; 1, position of amplicon relative to the 5′- end of mir-142 precursor sequence. (DOC) [file pone.0079231.s008.doc]

**Table S4 Overview of primers used for methylation-specific PCR and bisulfite sequencing.**

| **Primer name** | **Forward sequence (5’- 3’)** | **Reverse sequence (5’- 3’)** | **Tm (°C)** | **Amplicon** | **Size of amplicon (bp)** | **Positions^1^** |
| --- | --- | --- | --- | --- | --- | --- |
| **Methylation-specific PCR** | | | | | | |
| TSS_M_1_F and R | AAAACGCTCATCCCCTACGAACGAA | AGGGTTTATAATTTCGAGGTTACGT | 58 | MSP #1 | 95 | -1,318 to -1,224 |
| TSS_U_1_F and R | AAAACACTCATCCCCTACAAACAAA | GGGTAGGGTTTATAATTTTGAGGTTATT | 58 | MSP #1 | 99 | -1,318 to -1,228 |
| Pre_M_2_F and R | GGTAGTTTGAAGAGTATACGTCGAC | CAACAATAACGTAATCTCCGAA | 55 | MSP #2 | 146 | -34 to +112 |
| Pre_U_2_F and R | TTGGGTAGTTTGAAGAGTATATGTTGAT | CAACAATAACATAATCTCCAAAACC | 55 | MSP #2 | 149 | -37 to +112 |
| **Bisulfite sequencing** | | | | | | |
| TSS_BSP_1_F and R | GGAGTCAGGAGGCCTGGGCA | AGGGCAGCAGAGGAGCTGCT | 58 | Region #1 | 368 | -1,483 to -1,115 |
| Pre_BSP_2_F and R | GGGATTTTAGGAAGTTATAAGGAG | AACTACTATAACCTTTCCCCAACT | 55 | Region #2 | 366 | -95 to +271 |

Tm, annealing temperature; U and M, primers specific for unmethylated and methylated sequences; TSS, transcription start site; MSP; methylation-specific PCR; Pre, precursor sequence; ^1^, position of amplicon relative to the 5’- end of *mir-142* precursor sequence.
