# Supplementary material for: Epigenetic Regulation and Functional Characterization of MicroRNA-142 in Mesenchymal Cells
Source: PLoS One. 2013 Nov 13;8(11):e79231. doi: 10.1371/journal.pone.0079231 (PMC3827369; doi:10.1371/journal.pone.0079231)
Supplement: Table S5 — Overview of primers used to clone the putative regulatory region of the mir-142 gene. Tm, annealing temperature; 1, position of amplicon relative to the 5′- end of mir-142 precursor sequence. (DOC) [file pone.0079231.s009.doc]

**Table S5 Overview of primers used to clone the putative regulatory region of the *mir-142* gene.**

| **Primer name** | **Forward sequence (5’- 3’)** | **Reverse sequence (5’- 3’)** | **Tm (°C)** | **Size of amplicon (bp)** | **Positions^1^** |
| --- | --- | --- | --- | --- | --- |
| 142_F_2031minus and 142_R_1minus | ATCGGGGGTGGCATGCAGGT | TGTCTGTCCGTCGGCGTGTACTC | 60 | 2,031 | -2,031 to +1 |
|  |  |  |  |  |  |

Tm, annealing temperature; ^1^, position of amplicon relative to the 5’- end of *mir-142* precursor sequence.
